# Supplementary material for: Ice rule fragility via topological charge transfer in artificial colloidal ice
Source: Nat Commun. 2018 Oct 8;9:4146. doi: 10.1038/s41467-018-06631-1 (PMC6175946; doi:10.1038/s41467-018-06631-1)
Supplement: Supplementary file 2 — Description of Additional Supplementary Files [file 41467_2018_6631_MOESM2_ESM.pdf]

Description of Additional Supplementary Files

File Name: Supplementary Movie 1
Description: Dynamics of repulsive colloidal particles in a non decimated square lattice of double wells subjected to a perpendicular magnetic field of amplitude B = 15mT (experimental).

File Name: Supplementary Movie 2
Description: Colloidal dynamics in a decimated square lattice when subjected to a perpendicular magnetic field of amplitude B = 15mT (experimental).

File Name: Supplementary Movie 3
Description: Dynamics of repulsive colloidal particles in a decimated square lattice (ξ = 0:02) of double wells as the field is ramped up (numerical).

File Name: Supplementary Movie 4
Description: Dynamics of repulsive colloidal particles in a decimated square lattice (ξ = 0:06) of double wells as the field is ramped up (numerical).

File Name: Supplementary Movie 5
Description: Dynamics of repulsive colloidal particles in a decimated square lattice (ξ = 0:12) of double wells as the field is ramped up (numerical).

File Name: Supplementary Movie 6
Description: Dynamics of repulsive colloidal particles in a decimated square lattice (ξ = 0:22) of double wells as the field is ramped up (numerical).
